# Insights Into Persistent HIV-1 Infection and Functional Cure: Novel Capabilities and Strategies

**DOI:** 10.3389/fmicb.2022.862270

**Published:** 2022-04-27

**Authors:** Tram M. Ta, Sajjaf Malik, Elizabeth M. Anderson, Amber D. Jones, Jocelyn Perchik, Maryann Freylikh, Luca Sardo, Zackary A. Klase, Taisuke Izumi

**Affiliations:** ^1^Department of Biological Sciences, Misher College of Arts and Sciences, University of the Sciences in Philadelphia, Philadelphia, PA, United States; ^2^Office of the Assistant Secretary for Health, Region 3, U.S. Department of Health and Human Services, Washington, DC, United States; ^3^Department of Pharmacology and Physiology, Drexel University College of Medicine, Philadelphia, PA, United States; ^4^Department of Infectious Disease and Vaccines, Merck & Co., Inc., Kenilworth, NJ, United States; ^5^Center for Neuroimmunology and CNS Therapeutics, Institute of Molecular Medicine and Infectious Diseases, Drexel University of Medicine, Philadelphia, PA, United States

**Keywords:** human immunodeficiency virus (HIV), functional HIV cure, HIV latency, HIV persistence, kick-and-kill strategy, Block-and-Lock strategy, defective proviruses, immunotherapy

## Abstract

Although HIV-1 replication can be efficiently suppressed to undetectable levels in peripheral blood by combination antiretroviral therapy (cART), lifelong medication is still required in people living with HIV (PLWH). Life expectancies have been extended by cART, but age-related comorbidities have increased which are associated with heavy physiological and economic burdens on PLWH. The obstacle to a functional HIV cure can be ascribed to the formation of latent reservoir establishment at the time of acute infection that persists during cART. Recent studies suggest that some HIV reservoirs are established in the early acute stages of HIV infection within multiple immune cells that are gradually shaped by various host and viral mechanisms and may undergo clonal expansion. Early cART initiation has been shown to reduce the reservoir size in HIV-infected individuals. Memory CD4+ T cell subsets are regarded as the predominant cellular compartment of the HIV reservoir, but monocytes and derivative macrophages or dendritic cells also play a role in the persistent virus infection. HIV latency is regulated at multiple molecular levels in transcriptional and post-transcriptional processes. Epigenetic regulation of the proviral promoter can profoundly regulate the viral transcription. In addition, transcriptional elongation, RNA splicing, and nuclear export pathways are also involved in maintaining HIV latency. Although most proviruses contain large internal deletions, some defective proviruses may induce immune activation by expressing viral proteins or producing replication-defective viral-like particles. In this review article, we discuss the state of the art on mechanisms of virus persistence in the periphery and tissue and summarize interdisciplinary approaches toward a functional HIV cure, including novel capabilities and strategies to measure and eliminate the infected reservoirs and induce immune control.

## Introduction

Human Immunodeficiency Virus Type-I (HIV-1) is the infectious agent that causes acquired immunodeficiency syndrome (AIDS) and remains a global concern due to the lack of effective vaccines and curative strategies (No Author, 2006). Despite the ability of combination antiviral therapy (cART) to effectively suppress plasma HIV-1 levels in the majority of people living with HIV (PLWH), HIV-1 persists by the formation of latent reservoirs established very early during the acute phase of HIV infection (Chun et al., 1997; Finzi et al., 1997; Holkmann Olsen et al., 2007; Kousignian et al., 2008; Wong et al., 2019). Furthermore, HIV-infected cells can undergo proliferation and over 50% of latently infected cells observed after prolonged cART are the product of clonal expansion (Ananworanich et al., 2015; Brodin et al., 2016; Yucha et al., 2017; Abrahams et al., 2019; Leite et al., 2019). Upon the cessation of cART, this reservoir can be reactivated and eventually leads to virus rebound in the majority of PLWH (Siliciano et al., 2003). Virus persistence necessitates lifelong cART for the 37.7 million PLWH and is possibly the last unmet challenge toward HIV cure/remission strategies. Persistence depends on multiple factors that are hypothesized to involve complex virus-host interactions. So far, there are two people who have been cured of HIV-1 infection, and one person who is undergoing long-term remission from HIV (Hütter et al., 2009; Allers et al., 2011; Gupta et al., 2019; Peluso et al., 2019). All of them had intercurrent leukemia and underwent a bone marrow transplant from donors who had a homozygous 32 base pair deletion in the CCR5 gene (CCR5-Δ32). Homozygous carriers of Δ32 deletion are largely resistant to HIV-1 infection because the mutation prevents functional expression of CCR5 that is used as a coreceptor for HIV-1 to enter immune cells. This mutation is primarily found in northern Europe because CCR5Δ32 selective pressures were thought to be induced by an intense smallpox epidemic in that region (Klitz et al., 2001; Galvani and Slatkin, 2003). Thus, hematopoietic stem cell transplant is currently the only successful strategy for a cure, but it is high risk and not scalable. Therefore, novel HIV curative strategies are urgently needed. One promising approach to eliminate cells that evade cART is the “kick-and-kill” strategy, where latency reversal agents (LRAs) reactivate the reservoir, allowing for immune cell recognition and elimination of these latently infected cells (Hamer, 2004). In contrast, the “Block-and-Lock” approach aims to induce permanent transcriptional and epigenetic proviral silencing, thereby preventing viral reactivation (Kessing et al., 2017). However, HIV-1 latency is still challenging for functional cure and remission. CRISPR-mediated genome editing is another promising approach to excise and deactivate the integrated HIV-1 DNA (Ebina et al., 2013; Hu et al., 2014). The United States Food and Drug Administration (FDA) recently approved clinical trials with a CRISPR-based strategy for HIV eradication (NCT05144386). Finally, modulation of the immune system has also been considered to address persistent reservoir elimination.

This review article discusses the obstacles to HIV eradication and multiple challenges for a functional cure of persistent HIV-1 infection recently highlighted in our research topic (Sardo et al., 2021).

## Human Immunodeficiency Virus Persistence

### Cellular Reservoir

**FIGURE 1:**
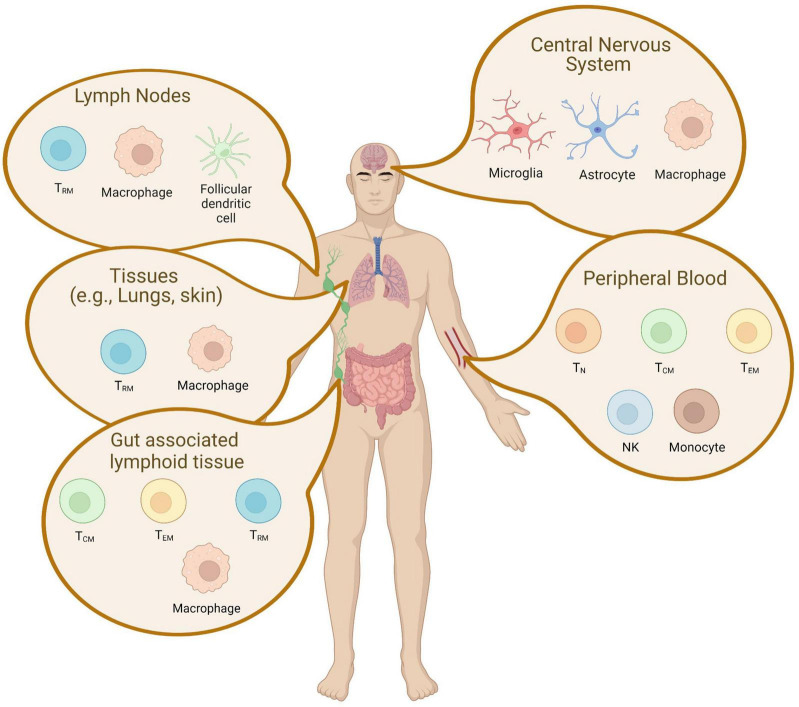
Latent cellular reservoir cells in tissue. A schematic representation of latently infected cells in tissues is depicted. The reservoirs are established in unique cell types and are localized across different tissues. CD4+ memory T cell subsets are found in the peripheral blood, the lymphoid tissue, gut-associated lymphoid tissue, and the central nervous system. This figure was created with BioRender.com.

The complexity and heterogeneity of HIV-1 reservoirs are caused by the establishment of latent infection into different cell types (Figure 1). HIV-1 primarily infects CD4+ T cells and the differentiation of T cells occurs in a stepwise fashion toward more differentiated cell types. The major cellular reservoirs for HIV-1 during cART reside in the memory CD4+ T cell subsets, especially central memory (T_*CM*_) subsets (Chomont et al., 2009; Buzon et al., 2014; Soriano-Sarabia et al., 2014; Ait-Ammar et al., 2020). Although having lower levels of integrated HIV-1 DNA due to their relative resistance to HIV-1 infection, the reactivation rate of latent proviruses in naive CD4+ T cells (T_*N*_) by LRAs is higher than in memory subsets (Ostrowski et al., 1999; Chomont et al., 2009; Wightman et al., 2010; Josefsson et al., 2013; Soriano-Sarabia et al., 2014). This suggests that the T_*N*_ subset might also be an important contributor to viral rebound in PLWH. Latently infected cells can ultimately differentiate into an effector memory subset (T_*EM*_), an activated phenotype involved in antigen responses (von Stockenstrom et al., 2015). T_*EM*_ harbors the largest proportion of intact-replication competent provirus (Hiener et al., 2017) and the largest inducible HIV-1 reservoir *in vitro* and *ex vivo* (Kulpa et al., 2019). Gamma delta (γδ) T cells that respond to non-peptide antigens through their distinct T-cell receptors bridge innate and adaptive immunity. The majority of γδ T cells in the blood are comprised of the Vδ2 subset, which also develops a memory phenotype. During HIV-1 infection, Vδ2 T-lymphocytes have been documented to be productively infected and depleted (Ait-Ammar et al., 2020). Vδ2 T cells isolated from HIV-1 infected individuals on prolonged cART contain replication-competent latent HIV-1 proviral DNA (Soriano-Sarabia et al., 2015). Although the memory subset of Vδ2 T cells has not been exclusively studied, peripheral Vδ2 T cells are also suggested to be a potential HIV-1 reservoir and should be targeted in curative approaches. While studies in the cellular reservoir have largely been conducted with the peripheral blood, memory subsets of CD4+ T cells containing HIV-1 sequence in gut-associated lymphoid tissue and lymph nodes (Tissue-resident memory T cells: T_*RM*_) were observed to contain 2–4 fold higher levels of HIV-1 DNA than the cells isolated from peripheral blood (Figure 1; Chun et al., 2008).

Great effort to characterize the persistent reservoir cells has been put into identifying markers that could help determine enriched latently HIV-infected cells (Darcis et al., 2019). Some of the markers, including HLA-DR, CD25, CD32, and CD69, are upregulated in the HIV-infected CD4+ T cells (Darcis et al., 2019). On the other hand, CD2 receptor has been found to be expressed on latently infected resting memory CD4+ T cells in virally suppressed individuals (Iglesias-Ussel et al., 2013). The expression of α4β7 integrin has been shown in a T cell subset that is highly susceptible to HIV-1 infection (Ding et al., 2015). Despite the extensive investigation to characterize latently infected CD4+ T cells, a unique and universal marker of the HIV-1 reservoir cells has not been identified yet.

Although CD4+ T cells are the most studied among cellular reservoirs in the peripheral blood, other targets such as cells of the myeloid lineage contribute to persistence. Myeloid cells are thought to be of great importance for the pathogenesis of HIV-1 in the central nervous system (CNS) (Figure 1). As the CNS contains very few CD4+ T cells, CNS reservoirs predominantly include both microglia and perivascular macrophages (Iglesias-Ussel et al., 2013; Ding et al., 2015; Cantero-Pérez et al., 2019; Darcis et al., 2019; Ait-Ammar et al., 2020). Both of these cell types appear to continue to express viral genes despite suppressive cART therapy (Colomer-Lluch et al., 2018; Cantero-Pérez et al., 2019). Like T-cells in the periphery, infected myeloid cells in a tissue can contribute to viral rebound (Gibellini et al., 2008; Ding et al., 2015; Gosselin et al., 2017) and the development of escape mutations (Guihot et al., 2018; Grau-Expósito et al., 2019). Specific to the CNS, these persistently infected myeloid populations are also central drivers of the neuropathologic, behavioral, and cognitive effects collectively known as neuroHIV (Ho et al., 2013; Wightman et al., 2015; Kandathil et al., 2016; Perlman, 2016; Gosselin et al., 2017; Yucha et al., 2017; Darcis et al., 2019; Wong et al., 2019; Zerbato et al., 2019). Myeloid cells have a longer lifespan than lymphocytes and can continue to produce viruses long after initial infection (Perlman, 2016). In addition, myeloid cells are resistant to HIV-induced apoptosis (Morison et al., 2005; Coppé et al., 2010; Perlman, 2016) and myeloid-derived macrophages are resistant against type I IFN inducible innate immunity, and Cytotoxic T-lymphocytes (CTL) mediated immune responses (Cantero-Pérez et al., 2019). This is distinct from infected T cell populations in the periphery (Rangarajan and Weinberg, 2003; Akhtar, 2015) that also become a predominant reservoir.

Laboratory mouse strains play a distinct role in biomedical research due to having many similarities of anatomy and physiology to humans and their ability to be genetically manipulated. However, mice cannot be infected with HIV-1, and thus steps must be taken to modify this usual model organism for use in HIV-1 experimental infections. Humanized mice engrafted with human hematopoietic systems have proven to be versatile experimental models for studying the fundamental aspects of HIV biology (Monod and Jacob, 1961; Rangarajan and Weinberg, 2003; Morison et al., 2005; Coppé et al., 2010; Bailey et al., 2013; Honeycutt et al., 2017). Both viral DNA and RNA were detected in CD4+ T cells and macrophages in bone marrow, liver, and thymus (BLT) transplanted mice (Honeycutt et al., 2017). The latent reservoir formation in either CD4+ T cells or macrophages was previously demonstrated to be independently developed using T-cell-only or myeloid-only humanized mice (Yucha et al., 2017). This suggests that both cell types are capable of establishing a persistent reservoir. HIV-infected macrophages circulate and infiltrate into various tissue compartments including the brain in human myeloid-only mice, suggesting that myeloid cells may seed deep tissue reservoirs. While most human macrophages have a rapid turnover of approximately 1 day in myeloid-only mice, viral rebound could be observed in the infected mice post ART interruption (Honeycutt et al., 2017), suggesting that specialized long-lived macrophages, such as microglia to persistent in the CNS, may contribute to viral reservoir formation over time. Another *in vivo* model, for instance non-human primates, is needed to further examine the relevance of myeloid-derived cells in HIV-1 reservoir formation.

### Mechanisms of Human Immunodeficiency Virus Type-I Latency

**FIGURE 2:**
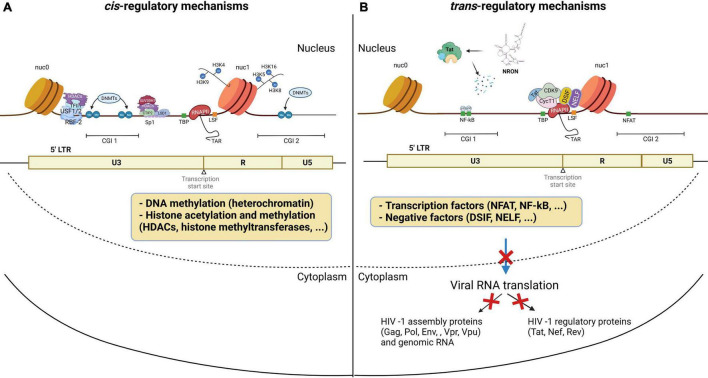
HIV-1 latency mechanisms. Latency is established through **(A)** cis-regulation elements, and **(B)** trans-regulation mechanisms predominantly regulate the HIV gene expression in the homeostatically proliferated reservoir cells at the chronic stage. **(A)** DNA methylation of the CG islands around the HIV-1 transcription start site maintains the HIV-1 promoter in a heterochromatic state to provoke HIV-1 transcriptional repression. Histone acetylation induces structural instability and increases access to transcriptional factors accessibility. **(B)** Transcription initiation is blocked by the low availability of NF-κB and phosphorylated NFAT at the promoter region. The binding of p50-p50 homodimers to the NF-κB binding site further inhibits transcription initiation. After initiation, RNA Pol II pauses at the promoter region due to the binding of negative elongation factors NELF and DSIF, leading to blocks in transcription elongation. In addition, P-TEFb is sequestered by the 7SK snRNP complex, causing a low expression level of Tat. The lncRNA called NRON degrades HIV-1 Tat to inhibit the P-TEFb formation. miRNAs targeting CyclinT1 regulate viral production and expression by overexpression in resting cells. This figure was created with BioRender.com.

Silent proviruses in infected cells are termed “latent” and are established and maintained, in part, by epigenetic modifications and by transcription factors and signaling molecules that reinforce latency (Van Lint et al., 1996; Triboulet et al., 2007; Sung and Rice, 2009; Mbonye and Karn, 2017). In fact, HIV-1 latency is mediated by both cis- and trans-regulatory mechanisms that prevent transcription factors from accessing the viral promoter region (Figure 2; Hakre et al., 2012; Donahue and Wainberg, 2013; Ruelas and Greene, 2013; Van Lint et al., 2013). Chromatin environments are dominantly cis-regulated, while viral and host transcription factors are trans-regulated in latently infected cells (Besnard et al., 2016). Cis-acting mechanisms depend on the chromatin environment at the virus integration site within the host genome (Figure 2A; Dahabieh et al., 2015). Cis-regulatory elements work through intracellular interactions between different parts of the same molecule, such as promoters, enhancers, and silencers (Goncalves et al., 2012). These elements are located in the vicinity of the genes they regulate and act as binding sites for transcription factors to regulate transcription rates of nearby genes (Davidson, 2021). DNA methylation, histone methylation, acetylation and crotonylation are commonly found in the viral promoter region as cis-regulatory mechanisms in latently infected cells. On the other hand, trans-regulatory factors cooperate with cis-regulatory elements to suppress viral gene expression (McManus et al., 2010; Goncalves et al., 2012; Wang et al., 2019).

Cellular epigenetic mechanisms to the cis-regulatory proviral regions have been extensively studied in peripheral CD4+ T cells. Clusters of CpG residues termed CG islands around the HIV-1 transcription site may be methylated, which would maintain the HIV-1 promoter in a heterochromatic state to repress HIV-1 transcription (Figure 2A; Blazkova et al., 2009; Kauder et al., 2009; Ruelas and Greene, 2013; Weber et al., 2014; Boltz et al., 2021). Histone acetyltransferases (HATs) are recruited to the viral long terminal repeat (LTR) and lead to increasing H3 and H4 lysine acetylation, specifically at H3K9, H3K4, H4K5, H4K8, and H4K16 residues (Benkirane et al., 1998; Hottiger and Nabel, 1998; Marzio et al., 1998; Col et al., 2001; Lusic, 2003). This acetylation leads to the relaxation of chromatin resulting in increased accessibility of transcription factors to the proviral DNA (Figure 2A; Mariño-Ramírez et al., 2005). Conversely, histone deacetylases (HDAC), remove acetyl groups from histones to promote chromatin condensation, thereby silencing proviruses (Jiang et al., 2007; Marban et al., 2007; Herbein and Wendling, 2010; Seto and Yoshida, 2014). Thus, HDAC inhibitors have been shown to potently reactivate latent cells *in vitro* (Saayman et al., 2014). In addition, H3 lysine methylations, including H3K4me, H3K36me, and H3K79me, are also involved in HIV DNA transcription and are mediated by histone methyltransferases such as SUV39H1 and EZH2 (du Chéné et al., 2007; Imai et al., 2010; Friedman et al., 2011; Ding et al., 2013; Tchasovnikarova et al., 2015; Li J. et al., 2016). Post-translational modification by lysine crotonylation was found to affect gene expression (Tan et al., 2011; Sabari et al., 2015, 2017). This study showed that histone crotonylation at the LTR through the induction of the enzyme acyl-CoA synthetase short-chain family member 2 (ACSS2) can reactivate latent HIV-1 *in vitro* and *ex vivo* (Jiang et al., 2018). Specifically, ACSS2 induction was found to increase histone crotonylation and acetylation at H3K4 while decreasing histone trimethylation at H3K27.

HIV-1 latency also results from the lack of trans-regulatory factors that enable T cell activation. Although many of these mechanisms are still under investigation, some transcription factors have been found to regulate viral gene expression. The positive transcription elongation factor, P-TEFb, is a critical kinase involved in HIV-1 transcription and is hijacked by the HIV-1 trans-activator, Tat (Figure 2B; Burnett et al., 2009). P-TEFb is sequestered by the 7SK small nuclear ribonucleoprotein (snRNP) complex, leading to an initial low expression of Tat (Weinberger et al., 2005). Once Tat is expressed, it associates with P-TEFb at the viral promoter to enhance HIV-1 transcription (Zhu et al., 1997; Yang et al., 2001). P-TEFb consists of CyclinT1 and CDK9 and requires CDK9 phosphorylation to activate the paused RNA Polymerase II (Pol II) (Wei et al., 1998; Ramakrishnan et al., 2009). CDK9 phosphorylation levels are generally lower in the resting CD4+ T cells, which is the dominant compartment of HIV-1 latently infected cells (Budhiraja et al., 2013; Ramakrishnan et al., 2015). A transcription factor, NF-κB, is also involved in the initiation of HIV-1 transcription (Adams et al., 1994; Williams et al., 2006). NF-κB is a host cell master regulator of inflammatory T cells that acts as a transcription factor to initiate HIV transcription in the absence of Tat (West et al., 2001). In resting cells, NF-κB is sequestered in the cytoplasm where it is bound by IκB. The low availability of NF-κB binding at the promoter region causes the change in the chromatin structure surrounding the LTR, leading to the disruption of the recruitment of Poll II and transcriptional initiation (Adams et al., 1994). Moreover, a mature NF-κB subunit, p50, can homodimerize to further suppress the NF-κB mediated transcription by binding to the HIV-1 promoter region and recruiting HDAC1 (Figure 2B; Zhong et al., 2002). Activated cytoplasmic PKC promotes ubiquitination and degradation of IκB to allow for nuclear translocation of NF-κB. The PKC pathway resulting in the activation of NF-κB is one of the most important pathways in HIV reactivation (reviewed in Colin and Van Lint, 2009; McKernan et al., 2012). PKC agonists, such as prostratin (Gulakowski et al., 1997; Williams et al., 2004), bryostatin-1 (Gutiérrez et al., 2016), and ingenol (Pandeló José et al., 2014), are highly effective in inducing latent HIV-1 expression from the viral reservoir through NK-κB signaling (Jiang and Dandekar, 2015). Therefore, PKC inhibitors have been investigated as promising candidates for HIV-1 eradication. In addition to the canonical NF-κB (cNF-κB) pathway, other NF-κB subpathways, including non-canonical (ncNF-κB) signaling, have been reported to regulate NF-κB responsible elements (Sun and Ley, 2008; Sun, 2017; Wong and Jiang, 2021). While cNF-κB signaling leads to RelA/p50 heterodimer binding to the promoter region (Deng et al., 2018), ncNF-κB signaling cleaves p100 into p52, which forms a transcriptional complex with RelB. Activation of ncNF-κB signaling was recently reported to induce HIV expression in both HIV-infected humanized mice and SIV-infected macaques under suppressive treatment (Hennessy et al., 2013; Nixon et al., 2020), indicating that ncNF-κB signaling is also involved in HIV latency.

Even after transcription initiation, RNA Pol II pauses at the promoter region due to the binding of negative elongation factors such as NELF and DSIF, causing RNA Pol II to terminate prematurely (Figure 2B; Yamaguchi et al., 1999). These mechanisms govern the HIV-1 promoter, tangling the LTR and preventing transcription factors from accessing the viral promoter region. Yukl et al. (2018) found that CD4+ T cells from PLWH on cART harbor more blocks to transcriptional elongation, completion, and splicing than transcriptional initiation. This indicates that the repression of HIV-1 transcription and splicing occurs at different stages and is regulated by different mechanisms, overall suggesting that HIV-1 latency is heterogeneous in CD4+ T cells.

Some of the proteins affecting viral transcription have been identified specifically in myeloid cells (Le Douce et al., 2012; Kruize and Kootstra, 2019), and interventions designed to block Tat function have been studied as a means to induce a latent state in macrophages and microglia (Alamer et al., 2020). Epigenetic regulation of HIV transcription does occur in myeloid cells, but studies that have examined changes in epigenetic modifiers at cellular promoters in response to HIV-1 infection in myeloid cells indicate that regulatory mechanisms are distinct from T cells (Wierda et al., 2012; Corley et al., 2016).

Non-coding RNAs (ncRNAs) have also been shown to be involved in gene regulation and mRNA splicing (Ranki et al., 1994), and were found to be involved in HIV-1 latency. Specifically, cellular miRNAs induce RNA silencing and post-transcriptional regulation of gene expression (Bissels et al., 2009). Long non-coding RNAs (lncRNAs) play key roles in RNA splicing and stability (Khalil et al., 2009). miRNAs modulate HIV-1 replication either by directly targeting the HIV-1 mRNA (Hariharan et al., 2005) or host mRNAs that code for transcription factors of HIV-1 (Brass et al., 2008; Zhou et al., 2008; Yeung et al., 2009; Houzet and Jeang, 2011). For instance, miR-17, miR-5p and miR-20a target PCAF (Triboulet et al., 2007), a transcriptional coactivator to interact with Tat and functionally synergize to activate the HIV-1 promoter (Mujtaba et al., 2002), and miR-198, miR-27b, miR-29b, miR-150, miR-223 target CyclinT1 (Sung and Rice, 2009; Chiang et al., 2012; Budhiraja et al., 2013) to regulate viral expression and production. Work by Carpio et al. (2010) and Klase et al. (2007) found that high expression of these miRNAs correlates with less susceptibility to HIV-1 infection in monocytes in comparison to macrophages. A set of mixed miRNAs enriched in monocytes represses the expression of PUR-α, a cofactor of Tat (Shen et al., 2012). The miRNA induced by Tat called miR-217 targets the host protein Sirtuin-1, which deacetylates and inactivates Tat, increasing HIV-1 expression (Zhang et al., 2012). A latency inducible lncRNA represented by NRON is highly expressed in resting CD4+ T cells and is involved in HIV-1 latency by mediating Tat degradation (Figure 2B; Li J. et al., 2016). Interestingly, the knockdown of NRON increases HIV-1 replication by enhancing NFAT activity (Imam et al., 2015). In another study, Li et al. reported that a novel lncRNA AK130181 (also named LOC105747689) was highly expressed in latently infected CD4+ T lymphocytes and inhibited HIV-1 transcription in an NF-κB-dependent manner (Li et al., 2020). Considering these latency mechanisms and other regulations for HIV-1 gene expression (Table 1), HIV-1 latency appears to be established by both cis- and trans-regulatory elements and in a cell lineage-specific manner.

**TABLE 1:** HIV latency mechanisms.

Mechanism of latency	Process	Factor(s)	Reference
Cis-regulation	DNA methylation	Heterochromatin	Blazkova et al., 2009; Kauder et al., 2009; Chávez et al., 2011
	Histone methylation	Histone methyltransferase	Chéné et al., 2007; Imai et al., 2010; Friedman et al., 2011; Boehm et al., 2017; Zhang et al., 2017; Huang et al., 2019
	Histone acetylation	HDACs	Lusic, 2003; Jiang et al., 2007; Marban et al., 2007; Tyagi and Karn, 2007; Li et al., 2018
	Histone crotonylation	ACSS2	Sabari et al., 2015; Jiang et al., 2018
Trans-regulation	Transcription initiation blocks	Low NFAT, Low NF-κB, Low STAT5, CTIP2, TRIM22, APOBEC3A	Williams et al., 2006; Marban et al., 2007; Della Chiara et al., 2011; Hakre et al., 2012; Turrini et al., 2015; Bosque et al., 2017; Forouzanfar et al., 2019; Taura et al., 2019
	Transcription elongation blocks	NELF, DSIF, Absence of Tat, Low P-TEFb	Yamaguchi et al., 1999; Nguyen et al., 2001; Yang et al., 2001; Weinberger et al., 2005; Burnett et al., 2009; Cherrier et al., 2013; Eilebrecht et al., 2014; Jadlowsky et al., 2014
	Post-transcriptional blocks	Low MATR3, PTB, PSF miRNAs (PCAF, CynclinT1) Long non-coding RNA (NRON)	Triboulet et al., 2007; Kula et al., 2011; Budhiraja et al., 2013; Suzuki et al., 2013; Rice, 2016; Kessing et al., 2017; Sarracino et al., 2018

### Human Immunodeficiency Virus Integration and Clonal Expansion

**FIGURE 3:**
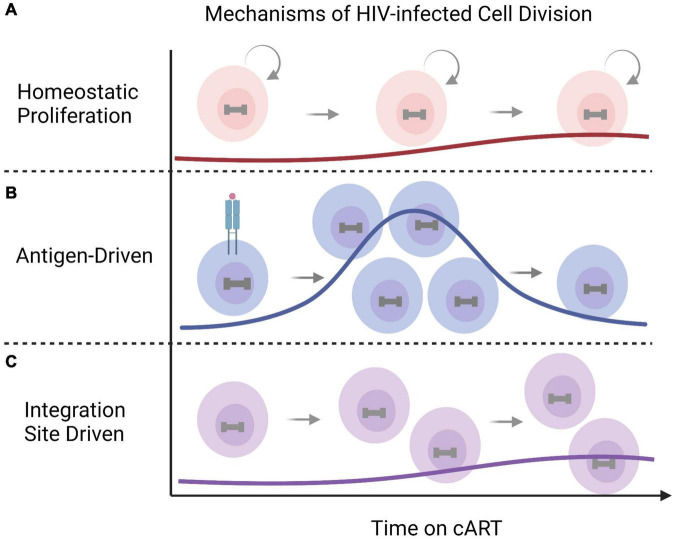
Mechanisms of clonal expansion in HIV-1 infected cells. HIV-infected cells undergo cell division overtime to maintain the HIV reservoir despite antiretroviral therapy. The mechanisms of HIV-1 infected clonal expansion include **(A)** normal homeostatic proliferation, **(B)** antigen-driven expansion in response to periodic or persistent cognate antigen exposures, or, in some cases, through **(C)** integration-site driven expansion (for example *BACH2*, *MKL2*, and *STAT5B)*. This figure was created with BioRender.com.

Integration is a hallmark of retroviruses and a central step in the HIV replication cycle that enables long-term HIV-1 persistence (Anderson and Maldarelli, 2018). HIV-1 integration into the host genome is generally non-specific with slight preferences toward introns of actively transcribed genes and typically exclude promoter regions (Mack et al., 2003; Han et al., 2004; Ikeda et al., 2007). cART successfully halts HIV replication by targeting various steps in the virus replication cycle, however current therapies do not target HIV-1 transcription or result in direct infected cell killing. The cells that harbor latent HIV-1 proviruses can persist for years despite therapy (Finzi et al., 1997; Wong et al., 1997; Siliciano et al., 2003) and undergo clonal expansion (Maldarelli et al., 2014; Wagner et al., 2014). The landscape of HIV-1 infected cells is shaped over time through cytopathic effects, immune responses, and infected cell proliferation (Anderson et al., 2020; Liu et al., 2020). HIV-1 infected cells can undergo clonal expansion through three distinct mechanisms: (1) homeostatic proliferation, (2) in response to the infected cells cognate antigen, or (3) in some cases as the result of the proviral integration site (Figure 3; Anderson and Maldarelli, 2018; Liu et al., 2020). Each driver of HIV-1 infected cell division can contribute to sustaining the HIV-1 reservoir despite cART. Homeostatic proliferation promotes HIV-1 persistence by allowing latently infected cells to undergo cell division in the absence of viral reactivation or cellular differentiation (Figure 3A; Chomont et al., 2009; Bosque et al., 2011; Musick et al., 2019). This indicates that the homeostatic proliferation of infected cells can sustain and replenish the HIV-1 reservoir while eluding the effects of cART. Antigen-driven clonal expansion occurs when an HIV-1 infected cell recognizes its cognate antigen (Figure 3B; Douek et al., 2002; Mendoza et al., 2020; Simonetti et al., 2021). HIV-1 primarily infects CD4+ T cells, which proliferate in response to antigenic interactions (Douek et al., 2002). The expansion of infected cells driven by antigen interactions is likely reflected in persistent, and/or waxing and waning of clonal populations through chronic and repeat exposures (Wang et al., 2018). Finally, the contribution of integration-site-driven clonal expansion likely plays only a minor role in sustaining the HIV-1 reservoir (Figure 3C) (Coffin et al., 2021). Proviral integration into oncogenes, such as *BACH2*, *MKL2*, and *STAT5B*, occurs rarely and is revealed after years of therapy, indicating the integration site itself may provide a selective advantage for long-term persistence (Maldarelli et al., 2014; Wagner et al., 2014; Cohn et al., 2015). All three mechanisms of HIV-1 infected cell proliferation can drive the persistence and expansion of the HIV-1 reservoir cells and pose major obstacles for curative strategies.

Although the majority of integrated proviruses are defective (Ho et al., 2013; Bruner et al., 2016, 2019; Imamichi et al., 2016), cells harboring intact replication-competent proviruses also undergo clonal expansion to sustain the HIV-1 reservoir despite cART (Simonetti et al., 2016; Bui et al., 2017; Lee et al., 2017; Patro et al., 2019). Once cART is stopped, viremia rebounds from this persistent, replication-competent reservoir to near pre-therapy levels within weeks for most individuals (Joos et al., 2008). It has recently been estimated that over 50% of inducible, replication-competent proviruses within the latent reservoir arose as the result of *in vivo* proliferation (Lorenzi et al., 2016; Bui et al., 2017; Hosmane et al., 2017). As this is likely an underestimate, these findings indicate that the proliferation of HIV-1 infected cells harboring replication-competent proviruses is one of the major mechanisms that maintains the reservoir and presents a challenge toward an HIV cure. Future curative strategies may aim to directly target cell-killing of HIV-1 infected cells or to blunt infected cell proliferation.

### Defective Proviruses

**FIGURE 4:**
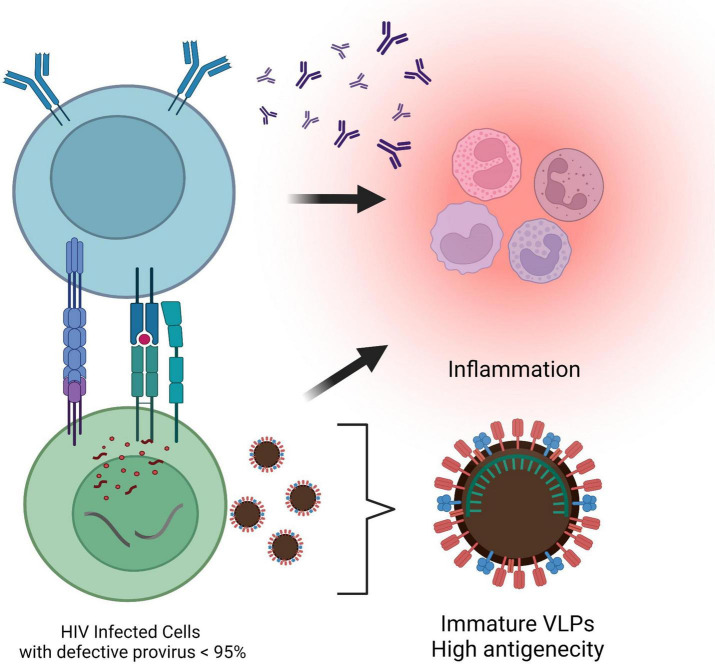
Antigenicity of the defective provirus. Most proviruses are defective with large internal deletions or hypermutations. Viral-like particles are released from novel unspliced viral RNAs transcribed from the defective proviruses. In addition, cytotoxic T lymphocytes are activated by the antigens presented by proviruses with defective major splice donors or hypermutations, which results in leading to chronic inflammation in individuals on cART. This figure was created with BioRender.com.

Although HIV-1 rebounds from replication-competent reservoir cells harboring intact proviral DNA upon cART cessation, the majority of proviruses are defective with large internal deletions or lethal mutations and clonally expand *in vivo* (Coffin and Hughes, 2021). Defective proviruses account for more than 95% of the total proviruses in the peripheral blood isolated from cART-treated PLWH (Siliciano et al., 2003; Eriksson et al., 2013; Crooks et al., 2015). Defective proviruses were initially thought to have little impact on HIV pathogenesis and disease progression due to their inability to produce infectious virions. However, it has recently been discovered that a fraction of defective proviruses are transcriptionally active (Imamichi et al., 2016) and can contribute to chronic immune activation (Pollack et al., 2017). Novel unspliced forms of viral RNAs transcription from defective proviruses can produce HIV-1 Gag and Pol proteins (Imamichi et al., 2020). These observations indicate that defective proviruses are capable of producing virus-like particles (VLPs) or viral proteins, which may become constitutive antigens (Figure 4). Chronic inflammation and persistent immune activation occurs in most individuals on long-term cART despite successful virus suppression and can induce immunological tolerance that enhances autoreactive antibody production, making it difficult to establish protective humoral immunity (van den Dries et al., 2017). VLPs have previously been employed for immunization purposes and demonstrated that immature morphology enhanced immunogenicity of VLPs and strongly induced IFN-γ secretion and antibody production (Álvarez-Fernández et al., 2012; Gonelli et al., 2019). Approximately 20% of the virions harboring intact protease still remain immature morphology after budding (Sarca et al., 2021), which implies that VLPs generated from defective proviruses might contribute to chronic inflammation in PLWH. In addition, proviruses with defective major splice donors or hypermutations induced by APOBEC3 cytidine deaminase enzymes (Izumi et al., 2008; Fukuda et al., 2019) can produce antigens against CTL and constitutively induce their activation (Shankar et al., 2000; Monajemi et al., 2014; Figure 4). It had long been thought that defective proviruses were irrelevant viral DNA sequences. However, defective proviruses capable of transcribing novel unspliced viral RNA can be found in individuals at all stages of virus infection, adding to the complexity of linked chronic immune stimulation in some PLWH.

## Cure Strategies

### Kick-and-Kill Strategy

**FIGURE 5:**
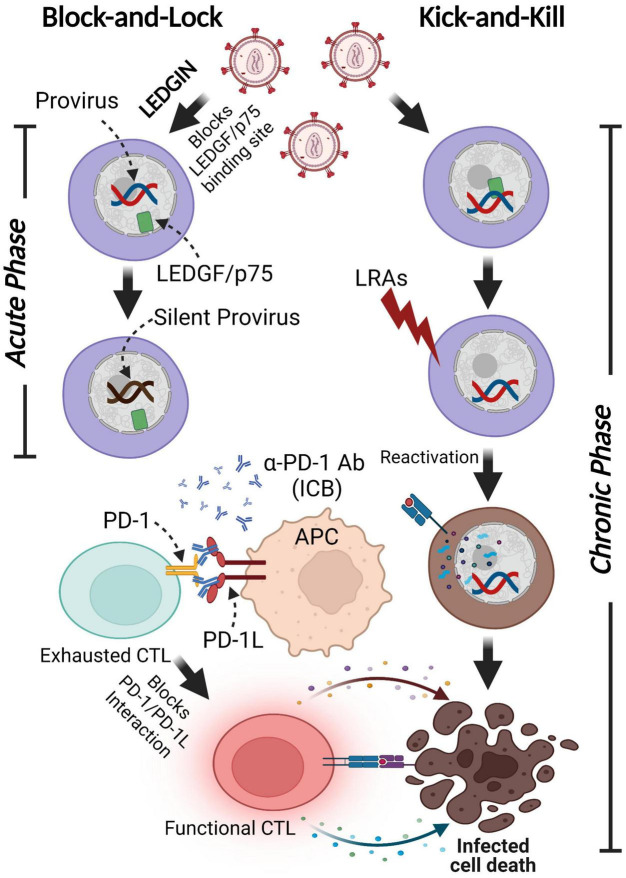
Functional cure strategies: Block-and-Lock and Kick-and-Kill approaches. A minor subset of infected cells harbors latent proviruses following infection. LEDGIN-mediated “Block-and-Lock” functional cure aims to permanently silence the provirus to block viral reactivation in the absence of cART. LEDGINs inhibit the LEDGF/p75-Integrase interaction, resulting in the redirection of integration into transcriptionally silent regions. The “Kick-and-Kill” strategy aims to decrease the size of functional HIV-1 reservoirs by reactivating proviral transcription with LRAs, leading to the elimination of infected cells via immune systems stimulated by ICB. This figure was created with BioRender.com.

One of the potential strategies toward HIV-1 eradication has been named “Kick-and-Kill” therapy. This approach is contingent upon the reactivation of latently infected cells in the presence of cART through the use of LRAs, and subsequent elimination of reactivated HIV-1 infected cells by the immune system or other interventions (Figure 5). Several pathways related to cis- and trans-regulating factors such as histone deacetylase (HDAC), NF-κB signaling, or PKC activation have been targeted to reactivate latently infected cells (Ait-Ammar et al., 2020). Several classes of LRAs have been described, to date (Table 2). Epigenetic modifiers such as histone acetylation/methylation, DNA methylation, or chromatin remodeling affect viral RNA transcription, splicing, or subsequent nuclear export pathway by altering the chromatin structure and DNA accessibility (Darcis et al., 2017; Ait-Ammar et al., 2020). HDAC inhibitors such as vorinostat, panobinostat, and romidepsin have been extensively studied in clinical trials, but the single-use of these inhibitors has not led to the eradication of latently infected cells *in vivo* (Kim et al., 2018). On the other hand, the combination of HIV-1 specific CD8+ cytotoxic T lymphocytes activation upon the usage of vorinostat showed promise to be effective for purging the latent reservoir cells in *ex vivo* experiments (Sengupta and Siliciano, 2018).

**TABLE 2:** LRAs classified based on their various activities.

LRA Class	Target	Drugs	References
Epigenetic modifiers	HDAC inhibition	HDACis: TSA, trapoxin, SAHA, romidepsin, panobinostat, entinostat, givinostat, valproic acid, MARK-1/11, AR-42, fimepinostat, chidamide	Van Lint et al., 1996; Quivy et al., 2002; Ylisastigui et al., 2004; Archin et al., 2009, 2012; Rasmussen et al., 2013; Wightman et al., 2013; Mates et al., 2015; Søgaard et al., 2015; Banga et al., 2016; Kuai et al., 2018; Yang et al., 2018; Gunst et al., 2019
	Suv39H1, G9a, SMYD2	HMTis: chaetocin, EPZ-6438, GSK-343, DZNEP, BIX-01294, UNC-0638	Friedman et al., 2011; Bouchat et al., 2012; Nguyen et al., 2017
	DNMT1, 3a, 3b	DNMTis: 5-AzaC, 5-AzadC	Bouchat et al., 2016
	RUNX1/STAT5	Benzodiazepines	Klase et al., 2014; Elbezanti et al., 2020; Lin et al., 2021
Benzotriazole derivatives	STAT5 activation	1-hydroxybenzotriazole (HOBt)	Bosque et al., 2017
Activators of Akt pathway	Upregulation of Akt	Disulfiram	Xing et al., 2011; Doyon et al., 2013; Spivak et al., 2014
	signaling pathway		
Inducers of P-TEFb release	Release of P-TEFb	BETis: JQ1, I-BET, I-BET151, OTX015, UMB-136, MMQO, CPI-203, RVX-208, PFI-1, BI-2536, and BI-6727 HMBA	Contreras et al., 2007; Bartholomeeusen et al., 2012; Lu et al., 2016, 2017; Huang et al., 2017; Abner et al., 2018; Gohda et al., 2018; Liang et al., 2019
SMAC mimetics	Induction of non-canonical	SBI-0637142	Pache et al., 2015; Hattori et al., 2018
	NF-κB pathways	Birinapant	
CCR5 antagonist	NF-κB activation	Maraviroc	López-Huertas et al., 2017; Madrid-Elena et al., 2018
MAPK agonist	Procyanidin trimer C1	MAP Kinase activation	Cary and Peterlin, 2018
PKC agonists	NF-κB activation	Prostratin Bryostatin-1 Ingenols: Ingenol-B, Ingenol 3,20-dibenzoate (Ingenol-db), ingenol-3-angelate (ingenol mebutate, PEP005)	Kulkosky et al., 2001; DeChristopher et al., 2012; Jiang L. et al., 2014; Spivak et al., 2014, 2015
Tat vaccine	Tat Oyi vaccine	Activation of HIV-1 LTR	Geng et al., 2016
	Tat-R5M4 protein		

PKC agonists may also be a suitable clinical approach for targeting NF-κB signaling in HIV-1 latency reactivation. Bryostatin, targeting NF-κB signaling, has been studied to reactivate latent cells *in vivo*, but unfortunately, no transcriptional enhancement was observed in HIV-1 latently infected cells in clinical settings (French et al., 2020). On the other hand, Derivatives of ingenol have been gaining interest in the reactivation of HIV-1 expression both *in vitro* and *in vivo* through NF-κB signaling (reviewed in Jiang and Dandekar, 2015). Ingenol-3-hexanoate was found to reactivate latent HIV-1 in J-Lat cells at a low concentration, hence lower cellular cytotoxicity (Jiang G. et al., 2014). Ingenol-3-angelate (PEP005), in combination with the P-TEFb agonist, JQ1, can synergistically reactivate latent HIV-1 with 7.5-fold higher effectiveness than PEP005 alone (Jiang et al., 2015). Interestingly, ACSS2-mediated histone crotonylation can also be associated with PEP005 to reactivate latent CD4+ T cells from HIV-infected individuals (Jiang et al., 2018). Ingenol 3,20-dibenzoate was found to activate resting CD4+ T cells from HIV-1 ART-treated aviremic patients and is potentially a marker to measure the reactivation of resting CD4+ T cells from treated PLWH (Spivak et al., 2015).

Recently, a family of IAP inhibitors (IAPi)/the mimetics of second mitochondria-derived activator of caspases (SMACm), such as Debio 1143 and AZD5582, have been proposed as a new class of LRAs *via* the induction of ncNF-κB signaling (Dashti et al., 2020). Debio 1143 was shown to lead to latent HIV reversal through the degradation of BIRC2/cIAP, a ubiquitin ligase that acts as a repressor of the ncNF-κB pathway *in vivo* using ART-suppressive BLT humanized mice or *ex vivo* using resting CD4+ T cells isolated from HIV-infected individuals with cART (Bobardt et al., 2019). AZD5582 was also found to induce SIV-RNA from latency in lymph node tissues of the ART-suppressive SIV-infected rhesus macaques by inhibiting BIRC2/cIAP. While these studies implied a potency of IAPi/SMACm as new LRAs *via* the novel ncNF-κB signaling pathway (Wong and Jiang, 2021), recent research applying AZD5582 failed to reduce reservoir size in the SHIV model (Nixon et al., 2020), indicating that latency reversal efficacy of IAPi/SMACm monotherapy may not be enough (Wong and Jiang, 2021). Furthermore, the induction of ncNF-κB signaling causes cell death in memory CD4+ T cells where latent HIV provirus is enriched (Wong and Jiang, 2021). Therefore, further studies are required to understand the complexity of both cNF-κB and ncNF-κB signaling pathways to create effective LRAs.

Other agonists targeting the innate immune receptors, TLR7 or TLR9, were also used to reactivate HIV-1 latent cells. The combination of HIV-1 Env-specific broadly neutralizing antibodies (bNAb) PGT121 together with the TLR7 agonist, vesatolimod GS-9620, delayed viral rebound following cART cessation in SIV/HIV chimeric virus (SHIV)-infected rhesus macaques that initiated antiretroviral treatment during acute infection (Bruno and Stoughton, 1984; Gutiérrez et al., 2016). However, the use of GS-9620 in phase I clinical trials with HIV-1 infected individuals on cART (NCT02858401) was discontinued due to adverse effects and an underwhelming impact on the plasma HIV-1 RNA and total DNA in CD4+ T cells. In addition, TLR9 agonist MGN1703 showed a moderate latency-reversing effect in HIV-1 infected participants, but a reduction in the reservoir size was not observed (Vibholm et al., 2017, 2019).

Considering that targeting a single mechanism with LRA monotherapy might not be sufficient to reactivate most latent cells, a combination of several different classes of LRAs targeting heterogenous silencing mechanisms may be required (Ait-Ammar et al., 2020). One study participant treated with MGN1703 showed viral control to undetectable levels upon cART cessation along with robust HIV-1 specific effector memory subset of CD8+ T cells and neutralization antibody production (Schleimann et al., 2019). This suggests that adaptive immunity is essential for the kick-and-kill approach. However, LRAs could impair multiple CD8+ T cell functions (Clutton and Jones, 2018). To date, successful reduction of the reservoir size *in vivo* has not been observed with LRA monotherapy, but has been seen using romidepsin and immune checkpoint blockade (ICB) nivolumab targeting PD-1 (Chun et al., 1997; Darcis et al., 2017).

As outlined above, the HIV-1 latent reservoir exists in cellular and tissue compartments and likely has multiple maintenance mechanisms. This may require diverse LRAs targeting a wide variety of latent cells, which poses challenges for therapies. Due to a lack of understanding of the various determinants contributing to the heterogeneity in HIV-1 latency, no LRA has been proved successful in clinical trials. Therefore, there is a need to understand the safety and potency of kick-and-kill strategies to develop effective LRA combination with immunostimulants to treat PLWH (Ait-Ammar et al., 2020).

### Immunomodulation

As we described before, HIV-1 preferentially infects CD4+ T cells and establishes long-term residency within affected individuals (Douek et al., 2002). In the standard progression of HIV-1 without therapy, normal progressors experience a decline in immune function (Boasso et al., 2009). Within the first 6–8 weeks post-exposure to HIV-1, the host experiences a significant decrease in CD4+ T cells, a concomitant increase in viral load, and a corresponding increase in immune activation (Fauci, 1991; Mellors et al., 1996; Coffin et al., 1997; Boasso et al., 2009) and a decrease in immune function. To control the infection, the host mounts an adaptive immune response via CD8+ T cells (Borrow et al., 1994; Koup et al., 1994). Despite these efforts, HIV persists in a small number of cells that can survive for a prolonged time.

Immune activation and inflammation are persistently driven by the ability of HIV-1 to evade detection and elimination by the immune system, the large-scale depletion of CD4+ T cells that regulate the adaptive immune response to infection, and the inability of cART to eliminate latently infected cells (Hazenberg et al., 2000; Gougeon, 2005; Leonard et al., 2008; Dinoso et al., 2009; Chun et al., 2010; Manel and Littman, 2011). During chronic infection, the host immune system must adapt to find new homeostasis which minimizes tissue damage from persistent inflammation while still maintaining control of infection. To mitigate immunopathology, the immune system has evolved mechanisms to progressively attenuate the immune response during chronic infection. Over time this leads to a state of hypo-responsiveness and eventual immunological tolerance of the virus. T cell exhaustion is one such mechanism in which effector T cells upregulate multiple inhibitory immune checkpoint receptors during chronic infection which results in the hierarchical loss of function (Xiong et al., 2001; Wherry et al., 2003; Saez-Cirion et al., 2007; McLane et al., 2019).

T cell exhaustion has been characterized by the co-expression of multiple inhibitory immune checkpoints such as cytotoxic lymphocyte antigen-4 (CTLA4), programmed cell death-1 (PD-1), T-cell immunoglobulin and mucin-domain-containing 3 (TIM3), T-cell immune receptor with Ig and ITIM domains (TIGIT), and lymphocyte-activation gene 3 (LAG3) on the surface of immune cells (Wherry and Kurachi, 2015). Immune checkpoints receptors, when engaged with their corresponding ligand, negatively regulate T cell activation (Blackburn et al., 2009). T cell exhaustion impacts the ability of CD8+ T cells to suppress viral replication (Mueller et al., 2001; Appay et al., 2002; Wherry et al., 2007; Wherry and Kurachi, 2015).

Recent literature has demonstrated that T cell exhaustion persists despite treatment with cART (Day et al., 2006; Nakanjako et al., 2011; Khoury et al., 2017; de Armas et al., 2019; Macatangay et al., 2020). While cART effectively reduces HIV-1 viral load to undetectable levels and significantly improves life expectancy, it is not a sterilizing cure nor does it fully restore host immune function (Migueles et al., 2009; Deeks, 2011; Arts and Hazuda, 2012; Wilson and Sereti, 2013; Serrano-Villar et al., 2014; Deeks et al., 2015; Perdomo-Celis et al., 2019). Poor immune reconstitution contributes to comorbidities that arise despite long-term treatment with cART, which necessitates an intervention that can restore an exhausted immune system (Baker et al., 2008). One such approach to rejuvenating exhausted T cells is through the use of immunotherapy in which monoclonal antibodies bind to immune checkpoints receptors, block their inhibitory signaling pathways, and consequently boost the immune response to chronic infection or cancer (Śledzińska et al., 2015). There are several FDA-approved cancer immunotherapies that target CTLA-4, PD-1, and PD-L1, which are now being investigated as potential novel strategies to reinvigorate a dysfunctional immune system exhausted by chronic HIV (Abbar et al., 2020; Sahin et al., 2020). The aspirational goal of such studies is to revitalize the immune system in such a way that PLWH could control the virus immunologically, without the need for intensive cART regimens.

The use of immunotherapy for PLWH was initially evaluated in HIV-1 positive cancer patients and it was determined that it was safe and effective for the treatment of cancer in patients with well-controlled viremia (Uldrick et al., 2017). Additional studies determined that the use of immunotherapy in these patients imposed no adverse effects on both CD4+ T cell count and plasma viral load (Cook and Kim, 2019; Abbar et al., 2020; Sahin et al., 2020), while some studies observed that participants experienced grade 3 or higher adverse events with various types of immunotherapy (Sahin et al., 2020). Guihot et al. (2018) demonstrated that treatment with PD-1 immunotherapy resulted in a dramatic decrease in plasma viral RNA and a concomitant increase in HIV-1 specific CD8+ T cells for a single cancer patient living with HIV (Guihot et al., 2018). The possible dual benefits of using immunotherapy to restore the functionality of exhausted CD8+ T cells, reverse HIV-1 latency through activation of viral transcription in CD4+ T cells (Wherry and Kurachi, 2015; Evans et al., 2018; Fromentin et al., 2019; Van der Sluis et al., 2020), and the favorable results of immunotherapy on HIV-1 positive cancer patients have led investigators to evaluate it for the treatment of virally suppressed otherwise healthy PLWH (Gay et al., 2017; Abbar et al., 2020). These studies concluded that participants were able to maintain viral suppression on immunotherapy, restored anti-HIV activity as demonstrated by a further reduction in plasma viral RNA varied (Gay et al., 2017; Abbar et al., 2020).

Immune checkpoint blockade has been utilized as immunotherapy to treat cells exhausted by chronic infection with cancer but these therapies have also been modestly effective at improving anti-tumor immunity (Pardoll, 2012; Shayan et al., 2017). One caveat to utilizing ICB for effective immunotherapy is the co-expression of multiple inhibitory receptors on immune cells (Shayan et al., 2017; Zahavi and Weiner, 2019) which might explain their modest impact at further reducing plasma viremia in otherwise healthy PLWH and warrants the use of combinatorial immunotherapy. Combinatorial therapy using ICB might not be sufficient to reverse the effects of exhaustion and restore the functionality of CD8+ T cells as recent studies have identified epigenetic modifications which may also restrict the effectiveness of ICB.

Epigenetic modifications are known to regulate T cell differentiation and are integral to the formation and heritability of various T cell subsets. These T cell subsets permit both the effective control of infection and also regulate effector function (Scharer et al., 2013; Chen et al., 2018; Henning et al., 2018; Zebley et al., 2020). Recent studies have begun to explore the epigenetic landscape of the exhausted CD8+ T cells and have determined that they are an epigenetically distinct subset of cells (Youngblood et al., 2011; Jadhav et al., 2019; Khan et al., 2019; Calle-Fabregat et al., 2020). Scharer et al. evaluated CD8+ T cell response to acute Lymphocytic Choriomeningitis virus infection from naïve and effector T cells and observed genome-wide DNA methylation of CD8+ T cells following T cell activation which permitted the inheritance of effector functions (Scharer et al., 2013). DNA methylation is a contributing epigenetic mechanism that regulates CD8+ T cell exhaustion (Youngblood et al., 2011, 2013; Scharer et al., 2013; Pauken et al., 2016; Ghoneim et al., 2017; Yates et al., 2021) and has been demonstrated to impact CD8 restoration by ICB (Ahn et al., 2016; Pauken et al., 2016; Ghoneim et al., 2017). It was demonstrated that T cell exhaustion can be categorized into two distinct stages which are delineated by *de novo* DNA methylation to the PD-1 promoter (Ghoneim et al., 2017). These epigenetic modifications to CD8+ T cells acquired during the effector phase regulate both the formation and heritability of terminally differentiated exhausted CD8+ T cells which preclude restoration by PD-1 blockade (Ahn et al., 2016; Pauken et al., 2016; Ghoneim et al., 2017). Epigenetic targeted therapy could potentially reverse the repressive epigenetic modifications that enforce CD8+ T cell exhaustion which could lead to a novel strategy in the treatment of HIV.

Chimeric antigen receptor (CAR)-T cell therapy is a kind of adaptive immunotherapy that genetically engineers a patient’s T cells to recognize and bind to foreign antigens on the antigen-expressing cells (Maus and Levine, 2016; Hartmann et al., 2017). Patients with hematologic malignancies, such as lymphocytic leukemia, lymphoblastic leukemia, diffuse large B-cell lymphoma, and follicular lymphoma, successfully achieved their treatment plans with CAR-T cell therapy (Porter et al., 2011; Maude et al., 2014; Schuster et al., 2017). Key components of CAR technology are the extracellular single-chain fragment variant derived from the antigen-binding region and the intracellular signaling domains containing CD3ζ, CD28, and 4-1BB (Maher et al., 2002; Imai et al., 2004; Porter et al., 2011; Srivastava and Riddell, 2015). Therefore, CAR can be designed to recognize specific antigens and subsequently induce activation of the immune response against target antigens. CD8+ T cells are collected from HIV-infected individuals and inserted with CAR genes *in vitro*, whose anti-HIV efficacy was verified, and then autologous HIV-specific CAR-T cells were transplanted into the patients (Qi et al., 2020). Recently, broadly neutralizing antibodies (bNAbs) targeting HIV-1 envelope glycoprotein have been used to construct anti-HIV specific CAR-T cells (Kwong et al., 2013; Ali et al., 2016). Hale et al. showed that CARs engineered with four types of bNAbs (PGT-128, PGT-145, VRC07-523, and 10E8) effectively activate and kill HIV-infected cells. Moreover, the integration of an HIV-1 CAR gene expression cassette into the CCR5 locus *via* homology-directed repair leads to the suppression of replicating of the virus (Hale et al., 2017). Currently, there are two ongoing clinical trials of CAR-T cell therapy in PLWH under cART (NCT03240328 and NCT03617198) to evaluate CD4-CAR T cells with CCR5 disruption for HIV resistance. Although there are obstacles in CAR-T cell therapy development, such as cell expansion *in vivo*, off-target effects, and severe cytokine storm (reviewed in Qi et al., 2020), it is worth exploring the potential of CAR-engineered T-cell therapy for an HIV cure.

Although current cART is very effective in targeting HIV pathogenesis, the pervasive nature of this disease requires the continued development of new ways to target viral replication and improve immune function. A focus on understanding the mechanisms of HIV-1 suppression by those with the innate ability to control the virus (Clerici et al., 1992; Kelker et al., 1992; Cao et al., 1995; Huang et al., 1995; Pinto et al., 1995; Rowland-Jones et al., 1995; Fowke et al., 1996; Lefrère et al., 1999; Shacklett, 2006; Dyer et al., 2008; Pereyra et al., 2008; Gonzalo-Gil et al., 2017; Pernas et al., 2018; Lopez-Galindez et al., 2019; Nguyen et al., 2019; Macatangay et al., 2020) has the potential to identify novel ways to improve host immune function, give insight into immune mechanisms that are common to both chronic infection and cancer, and rapidly treat PLWH with FDA approved therapies. As cells harboring reactivated proviruses by LRAs would then be required to be eliminated by CTL, the combination therapy with LRAs and ICBs/CAR-T could achieve a functional HIV-1 cure for chronic HIV infection (Figure 5).

### Block-and-Lock Strategy

The inability of the immune system to eradicate latently infected cells, due to the lack of viral protein expression, permits the long-term persistence of HIV-1 infected cells. In contrast to the “Kick-and-Kill” strategy, the “Block-and-Lock” approach aims to promote permanent provirus silencing even after cART cessation. This strategy ultimately seeks to affect both pre-integration and post-integration stages. HIV-1 DNA is preferentially integrated into transcriptionally active sites located near the nuclear pore, where chromatin is decondensed (Demeulemeester et al., 2015). Integration into a transcriptionally inactive site promotes proviral silencing. Lens epithelium-derived growth factor (LEDGF/p75) is a chromatin-binding host protein that supports HIV-1 DNA integration through interactions with the HIV-1 integrase protein (Vranckx et al., 2016). Treatment with a LEDGF inhibitor, LEDGINs, can dramatically redirect HIV-1 DNA integration sites to regions that are resistant to reactivation, potentially leading to a deeply silenced reservoir even after cART cessation (Figure 5; Christ et al., 2012; Kessl et al., 2012; Vranckx et al., 2016). LEDGIN treatment in the case of successful viral integration retargets the transcriptional factors out of active genes, which results in a prolonged latent state. This can lead to the inability of LRAs to reactivate silenced integrated proviruses (Gao et al., 2020). Therefore, LEDGINs might only help to reduce HIV-1 reservoir susceptibility to reactivation early after infection, prior to integration and the seeding of the reservoir.

A post-integration Block-and-Lock strategy aims to permanently suppress HIV-1 transcription to prevent viral reactivation even after successful proviral DNA integration. Post-integration silencing methods target the trans-regulation mechanisms to suppress viral gene expression by inhibiting viral and host transcription factors such as HIV-1 Tat, P-TEFb, and NF-κB. Tat is required for the stimulation of HIV-1 transcriptional elongation by binding an RNA element in the LTR and recruiting several transcription-activating proteins (Roy et al., 1990; Zhu et al., 1997). Didehydro-cortistatin A (dCA), the equipotent analog of cortistatin A, inhibits Tat-mediated transactivation through the interaction with the TAR domain of Tat (Li et al., 2019; Mediouni et al., 2019). Prior studies demonstrated that CDK9 inhibitors block viral transcription by disrupting P-TEFb formation (Pisell et al., 2001; Rice, 2016). In addition, activation of CDK2 that inhibits HIV-1 transcription and activation of the HIV-1 provirus through Tat phosphorylation was also targeted for the “Block-and-Lock” Strategy (Ammosova et al., 2006). Bisacetamide-induced protein (HEXIM-1) and 7SK small nuclear RNA interact and retain P-TEFb away from HIV-1 LTR (Yang et al., 2001; Yik et al., 2003). Bromodomain-containing protein 4 (BRD4) that competes with Tat for the P-TEFb interaction domain to further prevent HIV transcription is another target for permanent silencing (Jang et al., 2005; Bisgrove et al., 2007).

NF-κB is predominantly sequestered in the cytoplasm by IκB and cannot activate HIV-1 transcription in resting latently infected cells (Baeuerle and Henkel, 1994; Baldwin, 1996). Inhibition of NF-κB signaling is also considered as a Block-and-Lock strategy. Latent HIV proviruses were less reactivated by curaxin, a drug also used in immuno-oncology that inhibits NF-κB mediated transcription (Orphanides et al., 1998; Gasparian et al., 2011). This led to the hypothesis that curaxin could induce HIV latency via strengthening NF-κB inhibition.

Tat and NF-κB are required for HIV-1 gene expression, therefore inhibition of these critical components can lead to a post-integration “Block-and-Lock” strategy. The post-integration approach may necessitate PLWH to undergo life-long treatment to permanently suppress viral expression and frequently monitor viremia levels. Therefore, the combination of both methods, “Block-and-Kick-and-Kill” would lead to the functional cure for HIV-1 infection by reducing the reservoir size during acute infection through the redirection of HIV-1 DNA integration sites while subsequently reactivating the residual latently infected cells that are not deeply silenced, and eventually eliminate the reactivated cells by HIV-1 specific immune cells (Figure 5).

### Genome Editing Strategy

Genome editing technologies, such as the transcription activator-like nucleases (TALENs), zinc finger nucleases (ZFNs), and clustered regularly interspaced short palindromic repeat (CRISPR)-associated nuclease 9 (Cas9) have been proposed for novel approaches toward cure strategies. The use of Cas9 has recently been investigated with the advantages of precise insertion, deletion, and replacement of target double-strand DNA (dsDNA) (White et al., 2017). Cas9 has quickly become the preferred genome-editing platform for interrogating endogenous gene function *in vivo*. It was originally found in a bacterial adaptive immune defense system to play a vital role against DNA viruses or plasmids. Cas9 ribonucleoprotein complex consists of two components using endonuclease enzymes with a short-guide RNA (gRNA). Cas9 proteins are a specific class of enzymes that break the target dsDNA identified by gRNA, which is engineered with a particular sequence that guides the Cas9 protein to the target DNA sequence. Cas9 unwinds foreign DNA at sites complementary to the 20 base pair spacer region of the gRNA. If the DNA substrate is complementary to the gRNA, the Cas9 cleaves the invading DNA leading to gene inactivation. According to these unique features of Cas9 enzymatic activity, this system has been used as a genetic engineering technique to modify the genomes of living organisms.

Two people who had been living with HIV have been cured so far, the famous first case of the *Berlin patient* (Hütter et al., 2009; Allers et al., 2011), the second *London Patient* (Gupta et al., 2019), and a third individual, known as the *Dusseldorf Patient*, who is currently experiencing long-term remission (Peluso et al., 2019). These three individuals received a bone marrow transplant from matched donors with a homozygous 32 base pair deletion in the CCR5 gene (CCR5-Δ32) as part of their leukemia treatment. It has been documented that homozygous carriers of Δ32 mutation are largely resistant to R5 tropic HIV-1 infection that is exclusively detected in the transmitted founder viruses during the acute infection because the mutation prevents functional expression of CCR5, a coreceptor used by HIV-1 to enter immune cells (Cocchi et al., 1995; Samson et al., 1996; Biti et al., 1997). In addition, CCR5 gene editing of CD4+ T cells mediated by ZFN has been conducted clinically in HIV-1 infected individuals and was demonstrated to be safe (NCT00842634) (Tebas et al., 2014). Long-term CCR5 disruption in hematopoietic stem cells (HSCs) by the CRISPR/Cas9 system was achieved in a mouse model in 2017 to confer HIV-1 resistance *in vivo* (Xu et al., 2017). Although the other HIV-1 coreceptor, CXCR4, has also been targeted by Cas9, CXCR4 modified cells showed resistance against HIV-1 infection (Schumann et al., 2015). However, possible adverse side effects after CXCR4 disruption are of great concern as CXCR4 plays a vital role in hematopoietic cell development and thymic differentiation (Samson et al., 1996; Dar et al., 2006). To overcome these challenges, a combination of the Cas9 genome editing system and piggyBac transposase tools enabled the introduction of a point mutation, P191A, in the CXCR4 gene that specifically prohibits HIV-1 infection without disrupting CXCR4 receptor function (Liu et al., 2018). Although it should be noted that the virus rebound had not been observed in spite of the CXCR4-tropic virus existence in the Berlin patient, another case has been reported in a patient with allogeneic transplantation from a CCR5Δ32 donor, where a CXCR4-tropic virus rebounded after cART cessation (Kordelas et al., 2014). In addition, it has also been shown that HIV-1 can infect macrophages in a coreceptor-independent manner, leading to endocytosis of the virus (Gobeil et al., 2012). Therefore, targeting HIV-1 coreceptors by gene editing machinery should be considered carefully for HIV cure strategy.

**FIGURE 6:**
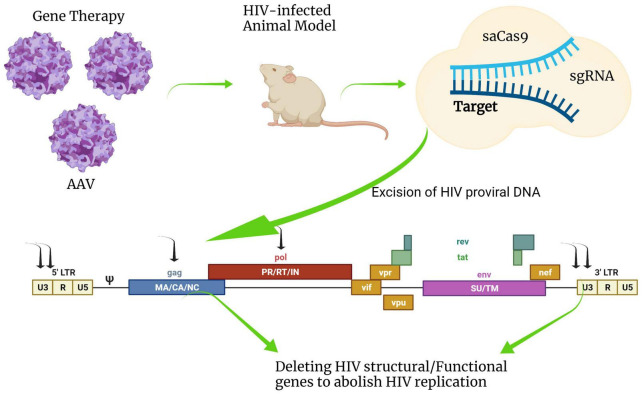
Schematic diagram of HIV-1 provirus DNA deactivation by CRISPR/Cas9 genome editing technology. Cas9 nuclease combined with gRNAs targeting multiple sites in HIV-1 DNA sequences such as 5′-LTR, 3′-LTR, gag, or pol can deactivate integrated viral DNA. Cas9 can be efficiently delivered by an adeno-associated virus vector (AAV) system *in vivo*. This figure was created with BioRender.com.

Cas9 could directly eliminate integrated proviral DNA *in vitro* by targeting the conserved sequence of the HIV-1 LTR U3 region in a latently infected T cell line, a monocytic cell line, and a microglial cell line (Hu et al., 2014). Recently, the excision of proviruses from latent reservoir cells was demonstrated *in vivo* in humanized mice by combining a provirus targeting genome editing tool with long-acting slow-effective antiviral therapy (Dash et al., 2019; Figure 6). Looking ahead to the future of CRISPR/Cas9 HIV treatment options, the U.S. Food and Drug Administration (FDA) has recently approved to begin trials testing EBT-101, an *in vivo* CRISPR/Cas9 gene therapy designed to excise HIV-1 proviral DNA. This is the first time the FDA has given investigational new drug (IND) approval to a CRISPR-based therapy for HIV treatment. Trials will evaluate the safety, tolerability, and efficacy of EBT-101 in healthy individuals living with HIV (NCT05144386).

Although many classes of viral vectors exist, the adeno-associated virus vector (AAV) has largely been used for delivering genome-editing machinery *in vivo* (Yin et al., 2017), and in clinical trials (NCT05144386). AAV is thought to be one of the most suitable viral vectors for gene therapy applications and gene transfer *in vivo*. AAV was approved for a number of human clinical trials in gene augmentation therapies due to its favorable safety profile. One major advantage of using AAV is a very mild immune response and toxicity elicited by AAV in animal models. However, AAV has some disadvantages, such as small cargo capacity, prolonged time for large-scale production, and relatively high cost. AAV has a substantial limitation in small viral genome packing capacity that is generally considered to be less than 5 kb, which is not suitable for large transgenes and is only available for Cas9 derived from smaller orthologs such as *Staphylococcus aureus (SaCas9)* or *Campylobacter jejuni* (CjCas9). The gRNA for Cas9 also requires a specific protospacer adjacent motif (PAM) that varies depending on the bacterial species. The most common Cas9 derived from *Staphylococcus pyogenes* (SpCas9) recognizes NGG directly downstream of the target sequence in the genomic DNA, while the PAM sequence of SaCas9 for optical target requires NNGRRT, limiting the design of specific gRNA target site. The length of the SpCas9 encoding gene is oversized to be packaged in AAV. The AAV-CRISPR system holds the potential to develop therapeutic options, but on the other hand, the development of a novel *in vivo* Cas9 delivery platform is urgently required to increase its flexibility.

## Human Immunodeficiency Virus Type-I Reservoir Detection

### Analytic Treatment Interruption

Accurately measuring the latent HIV reservoir is critical to assessing the effectiveness of curative strategies aimed at HIV remission. To date, the only way to definitively evaluate the effectiveness of curative strategies is with an analytic treatment interruption (ATI) in which the individual stops taking cART (Harari et al., 2012). The time that it takes for the viral rebound to occur after treatment cessation can be used to evaluate reservoir reduction. Theoretically, since viral rebound reflects the release of the virus from a stable reservoir, the smaller the size of the reservoir, the longer it takes for the viral rebound to occur (Hill et al., 2016; Li J. Z. et al., 2016). In the hopes to manage adverse effects associated with long-term use of cART, a clinical trial was performed in which HIV-infected individuals were randomly assigned to undergo either continuous suppressive cART or CD4+ count-guided episodic use of cART (NCT00027352) (El-Sadr et al., 2006). This study found that participants who undergo episodic treatment interruptions have significantly higher rates of opportunistic diseases or death from any cause when compared to participants on continuous cART. Moreover, the selection of drug-resistant mutations can occur during repeated treatment interruptions (Martinez-Picado et al., 2002), and rebounding viruses display increased IFNα2 and IFNβ resistance (Gondim et al., 2021). However, in some cases, individuals with high CD4 counts (>500 cells/ul) can safely undergo short CD4+ T cell count guided treatment interruptions without increased risk of morbidity or mortality and without developing drug resistance (Maggiolo et al., 2009; Routy et al., 2012; Ananworanich et al., 2015). While treatment interruption may provide evidence of complete viral eradication, individuals will most likely require long-term monitoring as unpredictable stochastic events can lead to viral rebound months to years later if the latent reservoir is not completely eradicated but only greatly reduced. Furthermore, the individual variability in time to rebound makes it hard to assess the magnitude of reservoir reduction as a result of eradication efforts.

### Quantitative Viral Outgrowth Assay

Currently, there is no accurate way to measure the latent reservoir *in vivo*. The quantitative viral outgrowth assay (QVOA) has been regarded as the “gold standard” for measuring the replication-competent latent reservoir size *ex vivo* (Finzi et al., 1997; Siliciano et al., 2003). QVOA measures the frequency of resting CD4+ T cells that produce infectious viruses after a single round of maximum global T cell activation. To this end, a large volume of resting CD4+ T cells are isolated from HIV-infected individuals and stimulated with the mitogen phytohemagglutinin (PHA) in the presence of uninfected γ-irradiated allogeneic PBMCs. Then the donor cells are co-cultured with either CD4+ T cells from a healthy donor or a cell line for 2–3 weeks before the infectious virus is measured in the culture supernatant by HIV-1 P24 ELISA (Finzi et al., 1997; Stuelke et al., 2020) or using a quantitative RT-PCR assay (Laird et al., 2013). However, some replication-competent viruses are induced only after multiple rounds of stimulation, indicating that standard QVOA may underestimate the size of the inducible latent reservoir (Abrahams et al., 2019). Furthermore, traditional QVOA is labor intensive and requires large amounts of sample. These limitations have led to the development of other approaches to estimate the size of the latent reservoir.

### PCR-Based Methods to Detect Human Immunodeficiency Virus Type-I Proviruses

Standard PCR-based techniques to quantify total HIV DNA are the easiest way to measure HIV-infected cells in PLWH. Total HIV DNA measurements inherently overestimate the size of the HIV reservoir since the majority of proviruses are defective or deleted (Bruner et al., 2016). However, total HIV DNA remains an important biomarker for viral persistence (Avettand-Fènoël et al., 2016), and levels of this marker are associated with viral rebound upon treatment cessation (Yerly et al., 2004; Williams et al., 2014). Recent approaches using droplet digital PCR (ddPCR) employ absolute quantification, which is more accurate than traditional quantitative PCR (qPCR) methods (Strain et al., 2013). Multiplexed ddPCR based assays have been used to track the fraction of deleted proviruses during cART (Anderson and Maldarelli, 2018; Anderson et al., 2020). Still, total HIV DNA measurements are at least two orders of magnitude higher than latent reservoir size measurements by QVOA (Eriksson et al., 2013). Total HIV DNA measurements may be further confounded by unintegrated HIV in either linear or 2-LTR circle forms, as these methods cannot distinguish integrated HIV DNA from non-integrated forms. Efforts to quantify only integrated HIV DNA utilize *Alu*-PCR (O’Doherty et al., 2002; Brady et al., 2013; De Spiegelaere et al., 2014). *Alu-gag-*PCR employs an outer forward primer that binds *Alu*, a repetitive element that is abundant in the human genome, and a reverse primer that is complementary to HIV *gag* DNA. This method will only amplify HIV proviruses integrated into the host genome and contain the primer target region of *gag* (O’Doherty et al., 2002; Liszewski et al., 2009). *Alu-gag-*PCR gives latent reservoir size estimates that are lower than total HIV DNA measurements but are still orders of magnitude higher than QVOA due to the inability of these assays to distinguish between replication-competent and defective proviruses (Eriksson et al., 2013).

Multiple methods have been developed to assess the proportion of intact versus obviously defective or deleted proviruses. Sequencing approaches such as matched integration site and proviral sequencing (MIP-seq) (Einkauf et al., 2019) and multiple-displacement amplification single genome sequencing (MDA-SGS) (Patro et al., 2019) can link full-length proviral sequences with their respective integration sites to infer replication competence as well as clonality. While these assays will provide great insights into the proviral landscape, the costs and labor required may hinder their use in large-scale studies. The intact proviral DNA assay (IPDA) is a high throughput ddPCR assay designed with two sets of primers located in conserved and frequently deleted regions of the viral genome (Bruner et al., 2019). IPDA offers a robust tool to estimate the number of intact proviruses and has been utilized as a surrogate for the latent reservoir (Simonetti et al., 2020). However, PCR failure as a result of primer mismatch due to HIV-1 diversity (Kinloch et al., 2021) and the inability to exclude proviruses that are defective or deleted in other regions (Gaebler et al., 2021) may preclude proper latent reservoir size measurements by IPDA. Other ddPCR strategies that utilize more target regions can further exclude defective proviruses. For example, a triplex digital PCR method (van Snippenberg et al., 2021) and a five-region approach that combines two triplex ddPCR assays (Levy et al., 2021) have been developed to help overcome the mischaracterization of intact proviruses while still utilizing a high throughput and relatively inexpensive digital PCR platform. The recently developed quadruplex PCR with four probes (Q4PCR) assay combines a four-probe qPCR strategy with near full-length sequencing to distinguish between intact and defective proviruses (Gaebler et al., 2019; Cho et al., 2022). In this assay, individual HIV genomes are amplified to near full length and are screened for the presence of 4 HIV targets with qPCR. Wells that are positive for at least two targets are then selected to undergo full-length proviral sequencing (Gaebler et al., 2019). Q4PCR is a lower-throughput assay compared to IPDA but allows for confirmation of proviral intactness. When compared head-to-head, IPDA and Q4PCR measurements correlated with one another but levels of intact proviruses measured with IPDA were approximately 19-fold higher than Q4PCR measurements (Gaebler et al., 2021). These differences in reservoir size estimates are likely from a combination of an overestimation by IPDA due to its inability to exclude defects in other regions of the provirus, and an underestimation by Q4PCR, due to inefficiencies from long-distance PCR. The actual number of intact proviruses may lie in between the measurements from IPDA and Q4PCR. Moreover, not all intact proviruses are replication-competent and ultimately give rise to rebound viremia. The chromatin environment at the proviral integration site as well as defects in transcription and/or translation of the provirus govern its ability to give rise to the infectious virus (Einkauf et al., 2022).

### Quantifying Functional Human Immunodeficiency Virus Type-I Proviruses

Strategies to more accurately estimate latent reservoir size aim to quantify replication-competent proviruses. The Tat/rev Induced Limiting Dilution Assay (TILDA) measures the frequency of cells harboring viral genomes that produce *tat/rev* multiply spliced HIV RNA (msRNA) upon maximum activation of CD4+ T cells (Procopio et al., 2015). The *tat/rev* msRNA is essential to produce infectious viruses (Pasternak et al., 2008). Many deleted proviruses lack *tat* and *rev* genes (Abrahams et al., 2019), and cells that contain *tat/rev* msRNA likely harbor a replication-competent infectious provirus. However, TILDA may still overestimate the actual size of the HIV reservoir since integrated proviruses that produce msRNA are not guaranteed to be infectious (Procopio et al., 2015). Recent advances in the development of next-generation *in situ* hybridization (ISH) technologies allow the detection of native viral DNA and RNA markers in histological specimens with greater sensitivity and faster workflow than traditional ISH (Deleage et al., 2016, 2018). These methods, namely DNAScope and RNAScope, can be leveraged to quantify the number of HIV-DNA and RNA-positive cells per gram of tissue (Estes et al., 2017). Uniquely among the methods described in this section, which are based on bulk sampling, tissue imaging approaches bypass the requirement of tissue homogenization and can provide spatial localization of the viral reservoir in tissue compartments. In addition, these allow multiplexing the detection of viral and host biomarkers to phenotype latently and actively infected cells and characterizing the complex heterogeneity of microenvironments that sustain virus persistence. However, these imaging-based approaches remain limited by the scarce accessibility of tissue specimens from clinical trials as opposed to blood and by the two-dimensional analysis of a few representative sections, which is based on the assumption that observations made can be inferred to the entire organ. In this regard, it is encouraging to see the emergence of total body positron emission tomography (PET) scanning (Santangelo et al., 2015; Henrich et al., 2019; Taylor et al., 2021) and whole organ imaging with novel tissue clearing technologies coupled with light-sheet microscopy (Tomer et al., 2014), which may represent new frontiers to detect viral reservoirs and broaden our understanding of virus persistence in tissue. Other approaches to identifying replication-competent reservoirs measure translation competent proviruses (Baxter et al., 2016). A flow-based RNA FISH assay simultaneously measures HIV RNA as well as Gag proteins upon phorbol 12-myristate 13-acetate (PMA) stimulation to identify single cells that are double positive for both cell-associated unspliced HIV *gag* RNA and its translation product Gag protein (HIV*^RNA+/Gag+^*). HIV*^RNA+/Gag+^* cells likely give rise to the infectious virus, enabling this assay to more accurately estimate the latent reservoir size compared to assays that viral RNA transcripts alone (Baxter et al., 2016). The advancement of digital enzyme-linked immunosorbent assay with a single molecule array (Simoa) has enabled the detection of cell-associated HIV gag p24 protein from both peripheral and tissue compartments with sensitivity higher than traditional ELISA (Passaes et al., 2017; Wu et al., 2017, 2021; Stuelke et al., 2020). These assays can be used to quantify steady-state and inducible reservoirs with the advantage of focusing on viruses capable of translating and processing Gag antigens that are relevant for infected cells clearance (Abdel-Mohsen et al., 2020). These further characterize reservoirs independently from their genetic intactness as defective proviruses can produce viral antigens and contribute to chronic immune system stimulation (Imamichi et al., 2020). Overall, rapid and accurate measurements of the true latent reservoir are needed to assess the effectiveness of curative strategies. For now, a combination of the described assays can be used to estimate the size of the latent reservoir, and the true size likely lies somewhere in between QVOA estimates and translation competent measurements.

## Summary

Once HIV infection is established, cART cannot eradicate the integrated proviruses due to latency establishment in multiple mechanisms (Table 1). HIV-1 gene transcription and subsequent translation are highly controlled by cis- and trans-regulatory elements and form distinct phenotypes of latent cells (Figure 2). HIV-1 reservoirs are generated in the early acute phase of virus infection with long-lasting treatment-resistant cells that can undergo clonal expansion during cART. Various strategies have been proposed to perturb the latent reservoir. The “Block-and-Lock” strategy aims to permanently silence the latent reservoir using latency-promoting agents such as LEDGINs. LEDGINSs can alter HIV-1 DNA integration sites to deeply silenced regions that are inefficient for reactivation even after cART cessation (Figure 5). While this treatment could be an approach to silence virus expression once and for all, it is not designed to have a profound effect on eradication after reservoir formation. Thus, the “Block-and-Lock” strategy could be effective when used during acute viral infection. The “Kick-and-Kill” strategy is designed to induce the expression of viral antigens by reactivation of latent cells (Figure 5). LRAs target both cis- and trans-mechanisms of the suppressive viral promoter to reactivate the latent cells, leading to the subsequent elimination by immune cells. Since individual LRA studies to date have ineffectively reduced reservoir size in clinical trials, several LRAs with distinct mechanisms of action may be needed to address the heterogeneity in latency.

While CD4+ T cells have been regarded as major cellular reservoir compartment, tissue localized long-lived myeloid cells may also have an essential role in HIV reservoir formation (Figure 1). The memory subsets of latently infected CD4+ T cells containing replication-competent proviral DNA are frequently detected in lymphoid tissue. In addition to the memory T cell subsets in peripheral blood and lymphoid tissue, macrophagic and astrocytic myeloid cells in the CNS pose a unique challenge for virus elimination (Figure 1).

During chronic infection, the immune system is in a state of non-reactivity to HIV-1 antigen caused by constant stimulation by viral antigens presentation or viral-like particles (VLPs) produced by defective proviruses that have large internal deletions or hypermutations (Figure 4).

The immune checkpoint blockade (ICB), which could either activate or inactivate specific immune cells could be useful to purge reactivated cells. The combination of LRAs with ICB has been demonstrated to effectively reduce the reservoir size *in vivo*, indicating that combinatorial approaches, in which LRAs are used to obtain a more effective shock and ICBs restore HIV-1 specific immune cells to eliminate reactivated reservoirs (Figure 5).

The Food and Drug Administration recently approved a human trial for HIV cure with CRISPR/Cas9 genome editing technology (Figure 6). While the adeno-associated viral vector (AAV) delivery system has several limitations, AAV is a valid option for *in vivo* Cas9 delivery to deactivate proviruses in tissue-localized reservoir cells in mice models. Cas9 targeting HIV-1 proviral DNA could potentially deactivate intact reservoir cells after the Kick-and-Kill therapy. Taken together, combining appropriate therapies at the stages of HIV acute and chronic infection, could contribute to new cases of cure without resorting to risky and unscalable bone marrow or stem cell transplantation.

## Author Contributions

TT, SM, EA, JP, MF, and TI draw figures. EA, LS, ZK, and TI designed the project. ZK and TI contributed to financial assistance. All authors listed wrote the manuscript and contributed to the article and approved the submitted version.

## Author Disclaimer

The findings of this article are those of the authors. They do not necessarily reflect the views of the Office of the Assistant Secretary for Health or the U.S. Department of Health and Human Services.

## Conflict of Interest

LS is employed by Merck Sharp & Dohme Corp., a subsidiary of Merck & Co., Inc., Kenilworth, NJ, United States. The remaining authors declare that the research was conducted in the absence of any commercial or financial relationships that could be construed as a potential conflict of interest.

## Publisher’s Note

All claims expressed in this article are solely those of the authors and do not necessarily represent those of their affiliated organizations, or those of the publisher, the editors and the reviewers. Any product that may be evaluated in this article, or claim that may be made by its manufacturer, is not guaranteed or endorsed by the publisher.
